# Recurrent epileptic seizures following cardiac catheterization with iodixanol: a case report

**DOI:** 10.1186/s12872-020-01341-3

**Published:** 2020-02-13

**Authors:** Pingping Lei, Weiping He, Quan Shi, Meiying Sun, Zhigang Sun

**Affiliations:** 1 Department of Cardiology, the Fifth Affiliated Hospital, Nanchang University, College of Medicine, Fuzhou, 344000 Jiangxi China; 2 Department of Neurology, the Fifth Affiliated Hospital, Nanchang University, College of Medicine, Fuzhou, 344000 Jiangxi China

**Keywords:** Contrast-induced encephalopathy, Epileptic seizures, Iodixanol, Cardiac catheterization

## Abstract

**Background:**

Contrast-induced encephalopathy (CIE) is a rare complication of cardiac catheterization; clinical manifestations include cortical blindness, seizures and focal neurological deficits. In general, recurrent epileptic seizures following cardiac catheterization with iodixanol occur more rarely than do other complications.

**Case presentation:**

Here, we report a case of a 76-year-old male patient who experienced unstable angina for nearly 10 months and was admitted to our hospital. Repeat cardiac catheterization was performed using iodixanol. At approximately 20 h after the first cardiac catheterization, his upper limbs began to exhibit slight trembling; the patient was conscious and could not control these movements. A total of 6 episodes occurred before the second cardiac catheterization was performed, with each episode lasting approximately 2 s. These symptoms were not treated. At approximately 2 h after the second cardiac catheterization, the symptoms became more severe, and the frequency of the episodes increased significantly; the symptoms had fully subsided at 6 h after the second operation. An electroencephalogram (EEG) demonstrated diffuse slowing with epileptiform abnormalities. Paroxysmal spike-wave and slow wave discharges were observed in the bilateral areas, and the abnormalities were marked in the frontal areas. These observations led us to conclude that the patient was experiencing epileptic seizures. During 6 months of monthly clinical follow-up visits after discharge, no abnormalities of the nervous system were found by cardiologists or neurologists, and the patient’s EEG was normal. No antiepileptic drugs were administered throughout this process.

**Conclusions:**

CIE, especially recurrent epileptic seizures, is a rare but often reversible complication of cardiac catheterization with iodixanol. Its symptoms can be mild and therefore are easily ignored by physicians. Early CIE detection may be achieved by EEG. Repeated exposure to contrast agents carries the risk of recurrent epileptic seizures.

## Background

Contrast-induced encephalopathy (CIE) due to the use of contrast agents is a rare complication of cardiac catheterization [1, 2]. The clinical manifestations of CIE include cortical blindness, seizures and focal neurological deficits [1, 2]. Although many factors have been considered as possible causes of CIE, current theories suggest a direct neurotoxic effect due to osmotic disruption of the blood-brain barrier [2]. In the present study, we report the case of a patient diagnosed with recurrent epileptic seizures after cardiac catheterization with iodixanol.

## Case presentation

A 76-year-old male patient of Asian descent who had experienced unstable angina for nearly 10 months was admitted to our hospital and was scheduled to undergo elective coronary angiography (CAG) a few days later. He had a history of hypertension, type 2 diabetes and transient ischaemic attacks (TIAs); he was diagnosed with type 2 diabetes 10 months previously but did not receive any treatment. He had smoked one pack of cigarettes per day for approximately50 years. There was no history of epilepsy in his family.

**Fig. 1:**
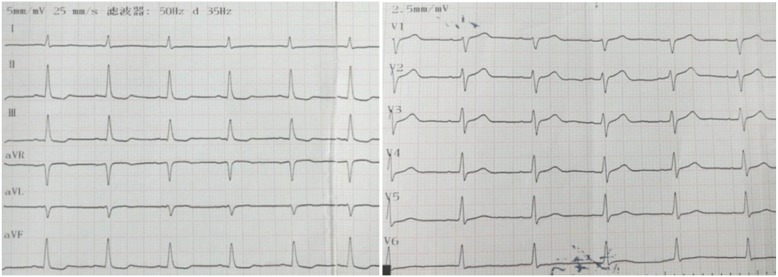
ECG showing ST segment depression in leads II, III and aVF

Physical examination revealed no pathologies; furthermore, no pathological results were obtained based on laboratory findings, and his cardiac enzyme levels (CK-MB, 1.17 ng/ml; troponin-I < 0.1 ng/ml) were normal. An electrocardiogram (ECG) revealed ST segment depression in leads II, III and aVF (Fig. 1). Transthoracic echocardiography demonstrated a normal left ventricular ejection fraction of 0.58.According to brain magnetic resonance imaging (MRI), mild stenosis of the cerebral arteries and anterior communicating artery aneurysms were present, with no recent cerebral ischaemia.

Four days after admission, cardiac catheterization was performed via the transradial approach. CAG revealed diffuse calcification from the proximal to the middle portion of the left anterior descending artery (LAD), resulting in approximately 90 and 60% stenosis in the left main coronary artery. The left circumflex artery (LCX) was very small. The right coronary artery (RCA) exhibited approximately 80% stenosis. However, because the coronary artery was severely calcified, the lesion could not be completely expanded. We suggested that rotational atherectomy (RA) should be performed by experts. For this procedure, a total of 80 ml of iodixanol (osmolality, 290 mOsm/kg H_2_O, which is the same as blood) was administered. At approximately 20 h after CAG, the patient’s upper limbs began to tremble slightly. The patient was conscious and could not control these movements. He did not call a physician, and no detailed examination was performed. A total of 6 episodes occurred during the 4 days before the second cardiac catheterization was performed. Each episode lasted approximately 2 s, and the episodes often occurred at night. As these symptoms were ignored, no treatment was administered.

**Fig. 2:**
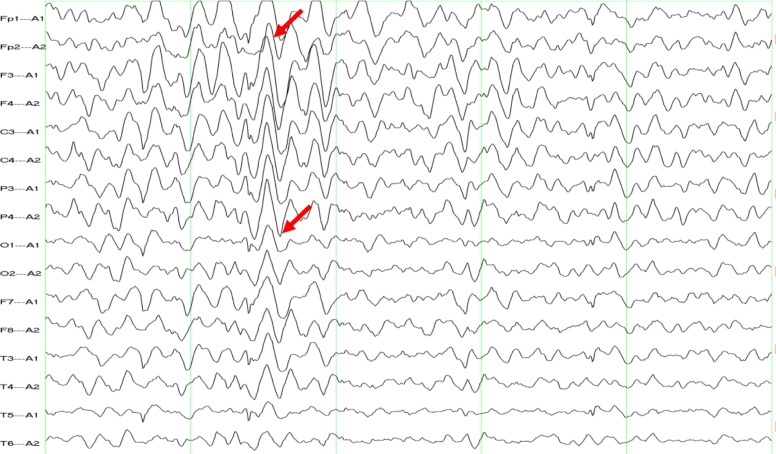
EEG demonstrating diffuse slowing with epileptiform abnormalities and paroxysmal spike-wave and slow wave discharges in the bilateral area

**Fig. 3:**
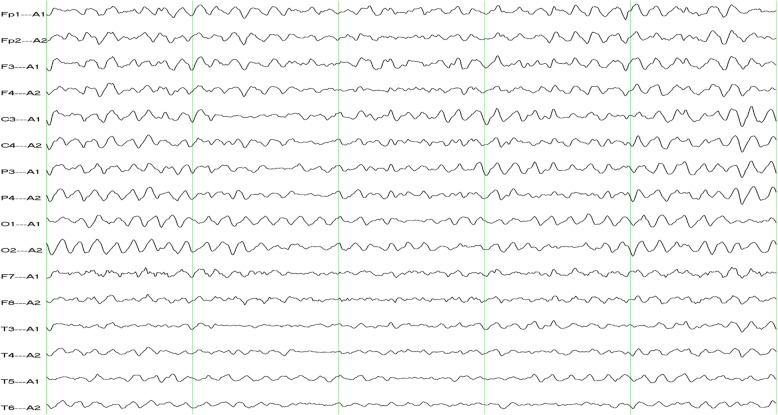
EEG showing normal results

Eight days after admission, RA was performed by an expert, and a 1.25-mm burr was gradually advanced into the lesion at 150,000 rpm until the burr finally passed through the lesion in the LAD. Following this procedure, two drug-eluting stents (DESs) were implanted in the distal (Firebird 2.75 × 33 mm, Microport) and middle (Firebird 3.0 × 33 mm, Microport) portions of the LAD. Another DES (Firebird 3.5 × 29 mm, Microport) was implanted in the proximal portion of the LAD and crossed over into the left main artery. Finally, another DES (Firebird 3.5 × 13 mm, Microport) was implanted in the middle portion of the RCA. The patient was manually administered 150 ml of iodixanol. At approximately 2 h after cardiac catheterization, his upper limbs again began to tremble slightly, and although he was still conscious, he could not control these movements. His symptoms subsequently became more serious, and the frequency of the episodes increased significantly. The patient’s neurological examination, vital signs, ECG, and serum glucose levels were normal; parameters related to liver and kidney function, electrolyte levels and thyroid function were also normal. His symptoms began to gradually attenuate 3 h later and had fully subsided at 6 h after the second operation. Neurologists recommended an electroencephalogram (EEG) examination, which was performed over two hours on the second day. The results showed diffuse slowing with epileptiform abnormalities; paroxysmal spike-wave and slow wave discharges were observed in the bilateral areas (Fig. 2), and the abnormalities were marked in the frontal areas (Fig. 2). The patient refused to undergo computed tomography (CT) or MRI. During 6 months of monthly clinical follow-up after discharge, no abnormalities of the nervous system were found by cardiologists and neurologists, and his EEG results were normal (Fig. 3). Throughout this process, no antiepileptic drugs were given.

## Discussion and conclusion

The most common known side effects of iodinated contrast agents after CAG include hypersensitivity reactions and contrast-induced nephropathy [1, 2]. CIE is a rare complication of cardiac catheterization and is often an acute reversible encephalopathy that occurs within minutes to hours after the procedure [1]. In our case, the patient’s upper limbs trembled slightly after the first operation, though these symptoms were ignored. His symptoms then became slightly more severe, and the frequency of the episodes increased significantly after the second operation. An EEG demonstrated diffuse slowing with epileptiform abnormalities. At 6 months after discharge, no abnormality was found in the patient’s nervous system, and his EEG results were normal; no antiepileptic drugs were administered. Although a cranial imaging examination was not performed, we believe that the patient experienced from CIE and epileptic seizures.

The mechanism by which contrast agents induce neurological injury has not been clearly elucidated. Studies have suggested that CIE occurs when the integrity of the blood-brain barrier is disrupted [1–3], allowing contrast medium to permeate the central nervous system, where it subsequently causes damage via direct neuronal toxicity [3]. Previous studies have also shown that iodine contrast agents may promote transient vasoconstriction [4]. In cases of CIE reported in the literature, the contrast media mostly consisted of high- and low-osmolality agents, such as iohexol, ioversol, and iopromide [1, 5–7]. Iso-osmolar contrast agents are not thought to cause CIE; however, cases of iso-osmolar contrast medium-induced CIE (iodixanol) have been reported, though they are rare [8–10]. In our case, the patient presented CIE after cardiac catheterization with iodixanol, yet the cause was unclear. The patient’s brain MRI showed mild stenosis of the cerebral arteries and anterior communicating artery aneurysms. We speculate that CIE may be related to anterior communicating artery aneurysms, and the integrity of the blood-brain barrier may have been much more easily disrupted than in other cases.

In the literature, CIE is often described as an acute and severe complication of cardiac catheterization [1, 2]. Brain CT and MRI are important for diagnosing CIE and can exclude thromboembolism and haemorrhage [2]. Characteristic CT findings include cortical or subcortical contrast enhancement, cerebral oedema, and hyperdensity in the subarachnoid space [1, 2]. Nonetheless, neuroimaging results maybe normal in some cases [11]. We did not perform CT or MRI in our study. The patient’s upper limbs trembled, indicating mild symptoms, but he refused to undergo CT or MRI because a brain MRI had been performed immediatelybefore the cardiac catheterization was performed. We consider the lack of neuroimaging a limitation of this study.

EEG abnormalities after cardiac catheterization have been reported [5–7, 12]. In some cases, EEG shows diffuse slowing. In our case, an EEG was performed after the patient’s symptoms disappeared, and the results demonstrated diffuse slowing with epileptiform abnormalities (paroxysmal spike-wave and slow wave discharges in the bilateral areas). Regardless, the times of appearance and disappearance of these abnormal EEG episodes are unknown in our case. Similarly, other studies have not indicated the time at which patients were diagnosed with CIE. Therefore, the duration of abnormal EEG results is worth exploring in future studies. Symptoms usually appear within minutes to hours after contrast administration, are serious, potentially including cortical blindness, seizures and focal neurological deficits, and are easy to identify and treat with interventions in the early stages [1]. In particular, Eleftheriou A et al. reported a patient who was diagnosed withlate CIE at approximately 22 h after CAG [10]. In our case, the symptoms were mild at first and were therefore ignored by doctors. A late diagnosis ofCIE is a dangerous situation for both patients and doctors. Therefore, if an EEG is performed immediately following cardiac catheterization, especially in patients with many risk factors for CIE, the associated abnormalities can be identified on the EEG results, and pre-intervention techniques can be performed to avoid adverse consequences. Thus, EEG may represent an early detection method for CIE.

Repeated exposure to contrast agents carries the risk of recurrent CIE. Law et al. reported a case in which the patient exhibitedrecurrent CIE and presented symptoms that manifested as homonymous hemianopia [9]. The patient had previously experienced a transient binocular vision disturbance following cardiac catheterization. Spina et al. also described recurrent CIE [13], whereby CIE manifested as transient limb weakness and aphasia following a first cardiac catheterization, and despite pre-medication with intravenous corticosteroids, aphasia occurred following the second cardiac catheterization. In our case, two coronary angiograms were performed, and the symptoms became more severe after the second cardiac catheterization. We suggest thatthe repeated use of these contrast agents likely worsened the patient’s neurologic symptoms and perpetuated the disease process. A previous study reported that patients who had a prior history of CIE and underwent repeat coronary angiogram with limited agent volumes and adequate hydration did not experience CIE [1]. Hence, if a patient has thoracalgia and must undergo repeated CAG, some interventions (such as pre-medication and the use of limited agent volumes and adequate hydration) can be performed to help avoid serious adverse reactions in the future.

In conclusion, CIE, especially recurrent epileptic seizures, is a rare complication of cardiac catheterization with iodixanol. CIE is often reversible. Its symptoms can be mild and are easily ignored by doctors. EEG may constitute a means of detecting CIE in the early stages. In general, repeated exposure to contrast agents carries the risk of recurrent epileptic seizures.

## Data Availability

All available information is contained within the present manuscript.

## Abbreviations

CAG: Coronary angiography
CIE: Contrast-induced encephalopathy
CT: Computed tomography
DES: Drug-eluting stent (DES)
ECG: Electrocardiogram;
EEG: Electroencephalogram;
LAD: Left anterior descending artery;
LCX: Left circumflex artery (LCX)
MRI: Magnetic resonance imaging
RA: Rotational atherectomy (RA)
RCA: Right coronary artery;
TIAs: Transient ischaemic attacks
